# Multiple dosing strategies with acetyl L-carnitine (ALCAR) fail to alter age-related hearing loss in the Fischer 344/NHsd rat

**DOI:** 10.1186/1477-5751-7-4

**Published:** 2008-07-11

**Authors:** Eric C Bielefeld, Donald Coling, Guang-Di Chen, Donald Henderson

**Affiliations:** 1 Center for Hearing and Deafness, Department of Communicative Disorders and Sciences, State University of New York at Buffalo, 137 Cary Hall, 3435 Main Street, Buffalo, NY 14214, USA

## Abstract

**Background:**

The Fischer 344/NHsd rat undergoes age-related, progressive, high-frequency hearing loss beginning at age 12 months. The loss has been linked to defects/death in the outer hair cells related to oxidative stress originating in the mitochondria. Acetyl L-carnitine (ALCAR) is known to enhance mitochondrial bioenergetics and membrane efficiency. Therefore, ALCAR was targeted as a possible pharmacologic intervention to prevent, or even restore, hearing loss from aging.

**Methods:**

Three different paradigms were used to deliver ALCAR to aging Fischer 344/NHsd rats. Rats in each condition had their hearing evaluated by auditory brainstem responses before, during, and after treatment. First, 24-month-old rats were given ALCAR (100 mg/kg dissolved 25 mg/ml in saline) by IP injection daily for one month. Second, 18-month-old rats were given ALCAR (100 mg/kg) by oral gavage for 90 days. Third, 15-month-old rats were given ALCAR (100 mg/kg) by oral gavage for 90 days. Control rats in each condition received saline by i.p. injection or gavage.

**Results:**

Hearing thresholds of the three sets of ALCAR-treated animals were never significantly different from their matched controls before, during, or after the treatments at any of the five test stimuli (5, 10, 20, and 40 kHz tone bursts and a click).

**Conclusion:**

The current study does not provide evidence that age-related hearing loss in the Fischer 344/NHsd rat can be altered with systemic administration of ALCAR.

## Background

The Fischer 344/NHsd (F344/NHsd) is an inbred, albino rat strain with a median life span of 28–31 months [1]. Its limited inter-animal variability makes it a useful candidate for the study of age-related hearing loss (ARHL). The F344/NHsd rat develops a progressive hearing loss that begins in the high frequencies and includes lower frequencies as the animal ages. The underlying pathology has been linked in part to a progressive loss of the outer hair cells (OHC), but the main contributor to the ARHL appears to be dysfunction of surviving OHC [2]. That OHC deterioration has been linked to oxidative stress. Age-related oxidative stress in the cochlea has been observed in a number of ARHL models [3-5]. Aging of the F344/NHsd rat has been linked to an accumulation of mitochondrial genetic deletions, suggesting the possibility that the mitochondria are a significant source of reactive oxygen species in the aging F344 rat cochlea [6,7]. The implication for ARHL was that increased reactive oxygen species in the OHC lead loss of OHC transduction due to metabolic insufficiency, with the long-term consequence of OHC death.

Because ARHL is such a pervasive, growing health problem [8,9], one of the ongoing research goals on ARHL is the development of intervention strategies to prevent, or potentially reverse, the progressive hearing loss. Caloric restriction and treatment with various antioxidants and pro-antioxidant molecules have delivered mixed results in various animal models [10-12]. Acetyl L-carnitine (ALCAR) is a molecule that enhances mitochondria membrane efficiency and bioenergetics, as well as having antioxidant properties. Seidman (2000) showed that treatment with ALCAR (300 mg/kg daily treatments) actually improved a group of F344 rats' hearing thresholds as they aged from 24 months to 26 months [10]. The implication was that improvement in mitochondrial efficiency not only led to reduced oxidative stress in the cochlea, but perhaps restored function to OHC that were alive but dysfunctional.

The working hypothesis for the current study was that long-term ALCAR treatment could prevent the loss of mitochondrial efficiency, which would lead to prevention of the loss of OHC function, leading to a reduction in the progression of ARHL in middle-aged (15–18 months) F344/NHsd rats, and/or restoration of some of the lost hearing in the middle-aged rats and rats of advanced age (24 months) that were already showing substantial hearing loss.

## Methods

A total of 44 F344/NHsd inbred male albino rats were used in the studies. They were obtained from Harlan Laboratories at 15 months-old (n = 18), 18 months-old (n = 18), and 24 months-old (n = 8). The minimum 15-month-old rats were chosen because they had already developed significant hearing loss 9 (10–20 dB in the 5–20 kHz range, 30–40 at 40 kHz [2]). The goal of the study was to restrict development of further hearing loss, and to possibly reverse some of the hearing loss already developed. Between 15 and 18 months, a loss of 10–20 dB across frequencies was anticipated. Between 18 and 23 months, a 15–30 dB loss was expected across frequencies. The animals were housed in a quiet colony (<45 dBA). All procedures involving use and care of the animals were reviewed and approved by the State University of New York at Buffalo Institutional Animal Care and Use Committee.

### Auditory Brainstem Response (ABR) testing

In order to assess the rate of hearing loss with age as well as the shape of the audiogram, the rats were tested for hearing sensitivity using free-field ABR thresholds. The animals were anesthetized with inhalant isoflurane (4% for induction, 1.5% for maintenance, 1 L/min O_2 _flow rate). Needle recording electrodes were placed at the vertex (non-inverting), below the left pinna (inverting) and behind the shoulder blade (ground). During ABR recording, the rats were placed on a homeothermic blanket to maintain body temperature. Test stimuli consisted of alternating phase tone bursts at frequencies of 5, 10, 20, and 40 kHz, as well as a click. Signals were generated using Tucker Davis Technologies (TDT, Gainesville, FL) SigGen software. Each tone burst (1 msec duration) was gated through a Blackmann window, and had a 0.5 msec rise/fall time with no plateau. The click had a 25 μsec duration, and was presented with alternating polarity. Stimuli were presented at a rate of 21/sec. Signals were routed to a Leaf tweeter (model AS-TH400A) positioned at zero degrees azimuth, 17 cm from the vertex of each rat's head. Acoustic stimuli were calibrated prior to each testing session, by recording the output of the speaker with a microphone placed at the animals' head level. The rats' evoked responses were collected in a 12.5 msec time window, and amplified with a gain of 50,000, using a TDT Headstage-4 bioamplifier, and bandpass filtered from 100–3000 Hz. 250 sweeps were averaged at each stimulus level using TDT BioSig software. The level of the signal was decreased in 5 dB steps from 90 dB pSPL to a level 15 dB below that of the lowest level that evoked a detectable and repeatable response. Threshold was recorded as the lowest level at which a detectable response was elicited and could be repeated.

### ALCAR treatments

In order to attempt to slow the progression of ARHL in the F344/NHsd rats, two experiments were run. In the first experiment, 15 month-old (n = 9 treated, 9 controls) were treated with ALCAR (100 mg/kg dissolved 25 mg/ml in sterile saline) by oral gavage once daily for 90 days. The control animals received saline by oral gavage. In the second experiment, 18 month-old rats (n = 9 treated, 9 controls) were treated for 90 days with ALCAR by oral gavage, with controls receiving saline.

In order to attempt to reverse some the already-established hearing loss in F344/NHsd rats of advanced age, eight 24 month-old rats (4 treated, 4 controls) were given ALCAR (100 mg/kg dissolved 25 mg/ml in sterile saline, pH adjusted to 7.2) by intraperitoneal (i.p.) injection once daily for 30 days. Control animals received i.p. injections of saline. The 100 mg/kg dose of ALCAR was chosen based on previous use of ALCAR to protect against noise-induced hearing loss in our laboratory and in a previous publication [12].

Weights and hearing status of all animals in the studies were compared to untreated, unhandled historical controls to assure that the gavaging process and the possible stress of daily handling did not alter the animals' aging process.

### Statistical Analysis

Three-factor ANOVAs (Treatment group × Time of test × Stimulus frequency) was used to analyze differences between the mean ABR thresholds of the different groups across the five different test stimuli (click, 5, 10, 20, and 40 kHz tone bursts). Treatment group and Frequency were analyzed as between-subjects variables. If a significant main effect occurred for Time of test or Frequency, post hoc testing with Tukey A tests was performed to delineate the nature of the differences. The three separate studies were analyzed separately from one another.

## Results

**Figure 1:**
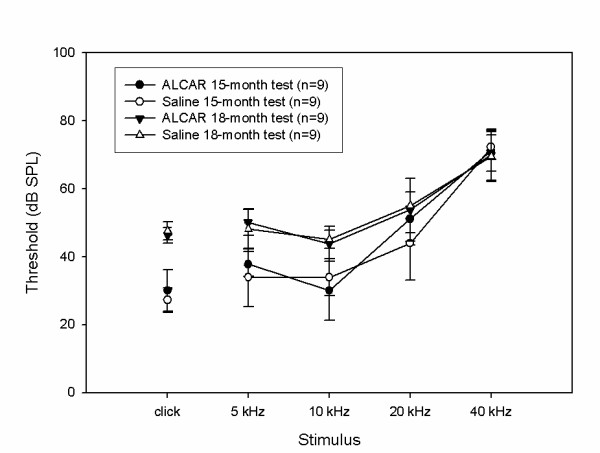
**Data from the study of F344/NHsd rats starting at 15 months of age.** Mean ABR thresholds in response to 5, 10, 20, and 40 kHz tone burst stimuli, as well as a click stimulus are displayed for rats aged 15 (dark and open circle symbols) and 18 months (dark and open triangle symbols). 9 animals were treated for three months with ALCAR (dark circle and dark triangle symbols); 9 were treated with saline as controls (open circle and triangle symbols). Error bars are +/- 1 standard deviation.

The first experiment was conducted with rats starting at 15 months of age (n = 9 treated, 9 controls). ABR thresholds were measured at 15 months (pre-treatment), 16, 17, and 18 months (final test after treatment concluded). Figure 1 displays the thresholds at the 15-month test and the 18-month test. Thresholds were compared between the treated and control groups at all four time points and across each test stimulus. Three-factor ANOVA revealed no significant interactions involving treatment group. A significant interaction of Time of test × Frequency was detected (p < 0.05), as the thresholds elevated by ~10–15 dB at 5 and 10 kHz and to the click, but thresholds at 20 and 40 kHz did not change over the 3-month treatment period. No main effects of treatment group were detected.

**Figure 2:**
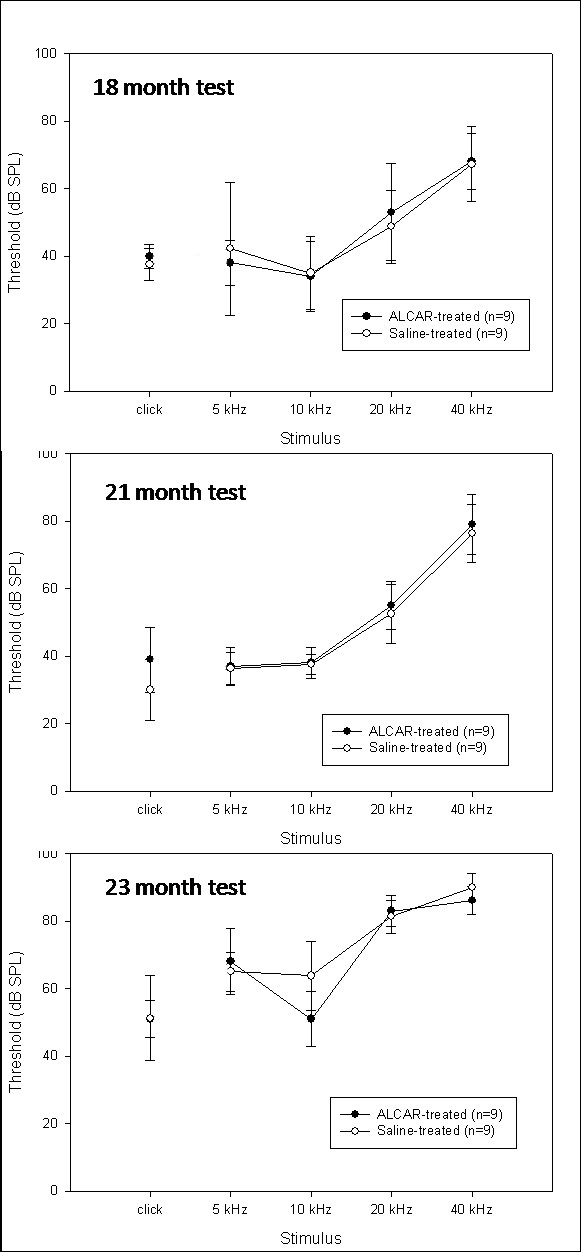
**Data from the study of F344/NHsd rats starting at 18 months of age.** Mean ABR thresholds in response to 5, 10, 20, and 40 kHz tone burst stimuli, as well as a click stimulus are displayed for rats aged 18 (Top Panel), 21 months (Middle Panel), and 23 months (Bottom Panel). 9 animals were treated for three months with ALCAR (dark circle symbols); 9 were treated with saline as controls (open circle symbols). Error bars are +/- 1 standard deviation.

The second experiment started the gavage ALCAR treatments in rats at 18 months of age (n = 9 treated, 9 controls). ABR thresholds were measured at 18 months (pre-treatment), 19, 20, 21 (final test after treatment concluded), 22, and 23 months. The 22 and 23-month tests were performed to assess whether the treatments had any delayed effects that would manifest after the treatment period had concluded. Figure 2 displays the thresholds at the 18-month test (Top Panel), 21-month test (Middle Panel), and the 23-month test (Bottom Panel). Three-factor ANOVA revealed no significant interactions involving treatment group. A significant interaction of Time of test × Frequency was detected (p < 0.05), as the thresholds elevated substantially over the 5-month observation period, though the extent of the changes varied with frequency. No main effects of treatment group were detected.

**Figure 3:**
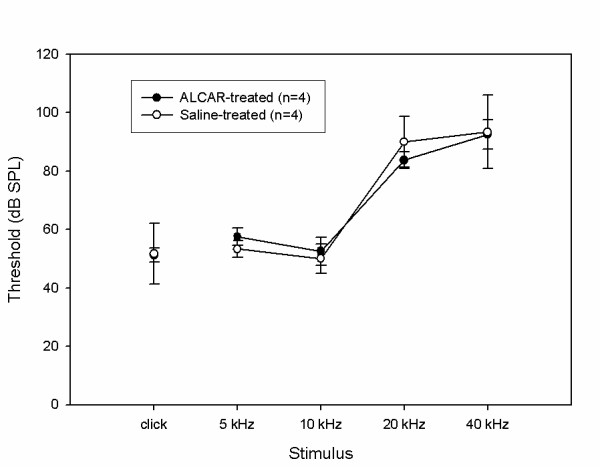
**Data from the study of F344/NHsd rats starting at 24 months of age. **Mean ABR thresholds in response to 5, 10, 20, and 40 kHz tone burst stimuli, as well as a click stimulus are displayed for rats aged 25 months. 4 animals were treated for one month from age 24 months to age 25 months with ALCAR (dark circle symbols); 4 were treated with saline as controls (open circle symbols). Error bars are +/- 1 standard deviation.

The third experiment used i.p. injections of ALCAR daily for one month, starting at age 24 months. ABR thresholds were measured at 24 months (pre-treatment) and 25 months (final test after treatment concluded). Figure 3 displays the thresholds at the 25-month test for the ALCAR-treated and saline-treated groups. Three-factor ANOVA revealed no significant interactions. A significant main effect Frequency was detected (p < 0.05), as the thresholds varied across stimulus frequency. No main effects of test time or treatment group were detected, indicating no changes in hearing over the one-month treatment period, nor any differences between the treated and untreated groups.

All saline-treated control groups were compared to age-matched untreated, unhandled controls from previous experiments. No differences were found between the saline controls and the unhandled controls for either the gavage or i.p. injection treatment paradigm, indicating no effects from the handling, gavaging, or i.p. injections.

## Discussion

The F344/NHsd rats showed no protective effects on their ARHL from the daily treatments with ALCAR, nor did the oldest animals show any reversal of their hearing loss from the more aggressive i.p. injection treatments. ALCAR was chosen because of the link between mitochondrial inefficiency and age-related OHC degeneration [6,7]. Additionally, ALCAR has been shown to protect the ear against noise-induced hearing loss [12-14], with the mechanism hypothesized to be improvement in mitochondrial efficiency leading to a reduction in noise-induced reactive oxygen species formation. Finally, ALCAR was used in a small study on the F344 rat's ARHL, and a small, but significant reduction in hearing thresholds was found in 24 month-old rats treated for 6 weeks with daily injections of 300 mg/kg ALCAR [10].

The lack of a demonstrated effect in the current studies is difficult to interpret, beyond the interpretation that the 100 mg/kg dose of ALCAR delivered to the F344/NHsd rat via the route (oral gavage and i.p. injection) on the dosing schedules that were employed is not effective in protecting against or reducing ARHL. Different doses, specifically a higher dose (300 mg/kg) that was used by Seidman (2000) may have been more effective. Different drugs, different animal models of ARHL, different dosing schedules all may prove to be more effective than those employed in the current studies. Specifically, starting treatment at 12 months of age may be effective, since high frequency (20 and 40 kHz) threshold appear to increase considerably between 12 and 15 months (comparing results from Bielefeld et al., (2008) in the 12 month-old rats to the current data on 15 month-old rats). It remains possible that systemic treatments did not permit enough ALCAR into the cochlea in order to influence cell survival, nor is it known whether the ALCAR treatments do indeed improve mitochondrial performance or reduce oxidative stress. Such questions will be the topics for future studies.

## Conclusion

While treatment with ALCAR is thought to improve OHC mitochondrial efficiency, thereby reducing oxidative stress in these cells, no benefits of treatment with ALCAR in aging F344/NHsd rats were detected. The goal of finding pharmacologic strategies to prevent or reverse ARHL remains a worthwhile topic that warrants further study.

## Abbreviations

ABR: auditory brainstem response; ALCAR: acetyl L-carnitine; ARHL: age-related hearing loss; F344/NHsd: Fischer 344/NHsd; i.p.: intraperitoneal; OHC: outer hair cell; TDT: Tucker Davis Technologies.

## Competing interests

The authors declare that they have no competing interests.

## Authors' contributions

EB carried out the ABR testing, participated in the ALCAR treatments, analyzed the data, and prepared the manuscript. DC and GD participated in the ABR testing and ALCAR treatments and were involved in designing the experiments. DH was involved in experiment design, interpretation of the results, and preparation of the manuscript. All authors read and approved the final manuscript.
